# Retrospective Analysis of Efficacy and Side Effects of Topical 4% Erythromycin Versus 1% Clindamycin Versus 20% Azelaic Acid During Pregnancy

**DOI:** 10.1111/jocd.70410

**Published:** 2025-08-28

**Authors:** Sabir Hasanbeyzade

**Affiliations:** ^1^ Dermatology and Venerology Department Hitit University, Erol Olçok Training and Research Hospital Çorum Türkiye

**Keywords:** acne, azelaic acid, clindamycin, erythromycin, pregnancy, treatment

## Abstract

**Background:**

Acne vulgaris is an inflammatory disease affecting the pilosebaceous unit, which also has psychological effects.

**Aims:**

Our aim in our study is to compare the recovery levels and side effect profiles of patients diagnosed with acne during pregnancy and who used topical erythromycin, clindamycin, or topical azelaic acid.

**Methods:**

After ethical approval was obtained for the study, the files of patients who applied to the outpatient clinic with acne complaints in 2018–2022 were retrospectively examined, and the files of 75 pregnant patients who used topical erythromycin, 96 who used clindamycin, and 26 who used azelaic acid were included in the study. Pre‐ and post‐treatment IGA values, lesion numbers, side effects during the treatment process, and patient satisfaction levels were examined.

**Results:**

When the groups were compared in terms of IGA value at the end of treatment and percentage improvements in all lesion numbers, it was seen that there was more improvement in the group using azelaic acid than the others (*p* < 0.001 for both). When the groups were compared in terms of side effects, no difference was found (*p* = 0.093). When the groups were compared in terms of satisfaction levels, there were significantly more patients who were very satisfied in the azelaic acid group (*p* < 0.001).

**Conclusion:**

As a result of the study, we see that azelaic acid is more successful in terms of both effectiveness and patient satisfaction. Therefore, azelaic acid is a good option in the treatment of mild to moderate acne during pregnancy.

## 1 Introduction

Acne vulgaris (AV) is an inflammatory disease of the pilosebaceous unit that can be seen at all ages, especially peaks in adolescence, and is characterized by comedones and inflammatory lesions in the form of papules and pustules. Hyperkeratinization, increase in seborrhea, Cutibacterium acnes colonization, and inflammatory processes play a role in its formation [1, 2]. Due to the increase in androgens during pregnancy, an exacerbation of acne may occur [3]. The psychological effects of acne are also known, and this situation becomes even more important during pregnancy, which is already accompanied by psychological fluctuations [4]. Although there are two main topics in drug treatment: topicals (benzoyl peroxide, retinoids, azelaic acid and antibiotics) and systemic treatments (antibiotics and isotretinoin), some of these options are not applicable during pregnancy. During pregnancy, category B macrolide group erythromycin, lincosamide group clindamycin, and topical anti‐microbial agent azelaic acid are among the drugs that can be used.

In this study, we retrospectively compared the effectiveness and side effect profiles of topical erythromycin (AKELA Forte 4% Gel, AKUR Sağlık Ürünleri Üretim Dağıtım ve Tic. A.Ş. Ümraniye/İstanbul/Türkiye), clindamycin (CLEOCIN‐T 1% topical solution, Pfizer PFE İlaçları A.Ş., Ortaköy/İstanbul/Türkiye, license number 223/18) and azelaic acid (AZELDERM 20% cream, ORVA İlaç San. ve Tic. A.Ş., Çiğli/İstanbul) used in the treatment of acne during pregnancy.

## 2 Materials and Methods

Ethics committee approval was obtained before the study. The study started by examining the medical records of patients who applied to the dermatology outpatient clinic with acne complaints between January 1, 2018, and December 31, 2022. Among these patients, patients who were pregnant and were started on topical erythromycin or clindamycin or azelaic acid as treatment were selected. Patients' identities have not been shared anywhere. Written informed consent was obtained from all patients before the study, and the study was conducted in accordance with the Declaration of Helsinki.

### 2.1 Exclusion Criteria

Patients who had previously received systemic isotretinoin treatment for AV, those who used systemic or topical corticosteroids at the time of admission, or those who had used them within the last 6 months were not included in the study. Patients who did not have a follow‐up period of at least 3 months after the start of treatment were also excluded from the study.

After all the eliminations, as a result, a total of 171 patients who applied to the dermatology outpatient clinic with acne complaints over a total period of 5 years were diagnosed with mild–moderate AV (IGA [Investigator's Global Assessment] score between 2 and 3), were pregnant at the time of admission, and were treated with topical erythromycin, clindamycin, or azelaic acid, and followed up for at least 3 months were included in the study. 75 of the patients were using erythromycin, 96 were using clindamycin, and 26 were using azelaic acid. Age, pre‐treatment and post‐treatment IGA values of the patients, the number of open and closed comedones separately, the number of papules and pustules (non‐inflammatory, inflammatory and total lesion numbers were also calculated), satisfaction status (very dissatisfied, satisfied and very satisfied) and information about the reasons why patients were dissatisfied were collected. The satisfaction levels of patients in their medical files were determined based on their own feedback. At the same time, information about whether side effects developed or not was collected from patient records too. At the end of 12 weeks of treatment, patients with an IGA value of 0 or 1 were evaluated as “clear” or “almost clear” respectively. The patients used the drugs twice a day, in the morning and in the evening.

### 2.2 Statistics

All data collected from patient files were included in the SPSS package program (IBM Statistics 25, USA), and analyses were performed. Discrete variables were expressed as numbers and percentages, and continuous variables were expressed as mean and standard deviation. The chi‐square test was used to determine whether there was a difference between groups in terms of discrete variables. The Kolmogorov–Smirnov test was used to check whether continuous variables followed a normal distribution, and those that did not follow a normal distribution in two independent groups (IGA values before and after treatment, all lesion numbers before and after treatment, improvement rates in IGA and all lesion numbers after treatment) were compared with the Mann–Whitney *U* test; those that did not comply with a normal distribution in three independent groups (age, IGA values before and after treatment, all lesion numbers before and after treatment, improvement rates in IGA and all lesion numbers after treatment) were compared with the Kruskal–Wallis test. The limit of statistical significance was determined when the alpha error was less than 5% (*p* < 0.05).

## 3 Results

There were 75 patients with a mean age of 29.96 ± 3.40 years (min. 23, max. 37) in the group using erythromycin, 96 patients with a mean age of 27.49 ± 3.32 years (min. 21, max. 35) in the group using clindamycin, and 26 patients with a mean age of 26.38 ± 2.4 (min. 24, max. 36) in the group using azelaic acid. There was no statistically significant difference in age between the groups (*p* = 0.257).

There was no significant difference between the groups in terms of initial IGA values, the number of non‐inflammatory lesions including open and closed comedones, and the number of papules and inflammatory lesions (*p* = 0.229, 0.718, 0.905, 0.488, 0.587 and 0.054, respectively). There was a significant difference in terms of the number of pustules and total lesions between the three groups (the number of pustules and total lesions were higher in the group using clindamycin, *p* = 0.046 and 0.023, respectively). When the groups were compared in pairs, it was seen that the group using clindamycin differed from the group using erythromycin in terms of the number of pustules and total lesions (*p* = 0.032 and 0.011, respectively), as well as the number of inflammatory lesions, and inflammatory lesions were more in this group (*p* = 0.019). The data are detailed in Table 1.

**TABLE 1:** Demographic characteristics, pre‐ and post‐treatment IGA values, lesion numbers and percentage improvements according to groups.

Mean ± SD	Erythromycin (*n* = 75)	Clindamycin (*n* = 96)	Azelaic acid (*n* = 26)	*p*
Age (years)	29.96 ± 3.40	27.49 ± 3.32	26.38 ± 2.4	0.257
Initial IGA values	2.36 ± 0.48	2.31 ± 0.72	2.58 ± 0.70	0.229
Inıtıal NNL	13.37 ± 3.08	13.70 ± 3.93	13.77 ± 6.04	0.488
*Open comedone*	*11.45* ± *3.18*	*11.80* ± *3.66*	*11.77 ± 4.96*	*0.718*
*Closed comedone*	*1.92* ± *1.57*	*1.91* ± *1.55*	*2 ± 1.26*	*0.905*
Inıtıal NIL	24.71 ± 7.23	26.91 ± 7.06	24.92 ± 5.18	0.054
*Papule*	*13.92* ± *1.61*	*13.73* ± *1.58*	*13.54 ± 2.47*	*0.587*
*Pustule*	*10.79* ± *6.73*	*13.18* ± *7.20*	*11.38 ± 3.01*	***0.046***
Inıtıal TNL	38.08 ± 7.73	40.60 ± 7.28	38.69 ± 10.65	**0.023**
EOT IGA values	1.61 ± 0.71	1.74 ± 0.63	0.77 ± 0.59	**< 0.001**
EOT NNL	13.84 ± 4.43	13.5 ± 4.23	9.12 ± 3.87	**< 0.001**
*Open comedone*	*11.43* ± *3.56*	*11.65* ± *4.04*	*8.42 ± 3.70*	***0.003***
*Closed comedone*	*2.41* ± *2.79*	*1.85* ± *1.94*	*0.69 ± 0.97*	***0.004***
EOT NIL	20.36 ± 8.89	25.61 ± 23.56	9.15 ± 5.66	**< 0.001**
*Papule*	*10.71* ± *4.82*	*12.67* ± *23.13*	*6.23 ± 4.3*	***< 0.001***
*Pustule*	*9.65* ± *6.8*	*12.95* ± *7.04*	*2.92 ± 2.71*	***< 0.001***
EOT TNL	34.20 ± 10.02	39.11 ± 23.68	18.27 ± 7.59	**< 0.001**
IGA IP	30 ± 33.22	28.29 ± 49.09	68.27 ± 23.1	**< 0.001**
Open comedones IP	0.6 ± 14.87	0.96 ± 20.91	25.35 ± 34.32	**< 0.001**
Closed comedones IP	−47.17 ± 186.55	−19.16 ± 142.56	67.05 ± 53.24	**< 0.001**
NNL IP	−2.98 ± 18.98	0.20 ± 24.62	30.19 ± 28.81	**< 0.001**
Papules IP	22.57 ± 34.81	6.72 ± 165.40	56.12 ± 28.74	**< 0.001**
Pustules IP	15.35 ± 17.43	0.68 ± 20.38	74.57 ± 24.17	**< 0.001**
NIL IP	18.97 ± 24.15	2.56 ± 120.49	64.34 ± 20.82	**< 0.001**
TNL IP	10.74 ± 15.89	3.39 ± 60.1	53.27 ± 16.21	**< 0.001**

*Note:* Bold letters in the table indicate significant results.

Abbreviations: EOT, end of treatment; IP, improvement percentage; NIL, number of inflammatory lesions; NNL, number of noninflammatory lesions; TNL, total number of lesions.

Then, it was examined whether there was a statistically significant difference between the 3 groups in terms of post‐treatment IGA values and individual lesion counts. A difference was found between the groups in terms of IGA values and all lesion counts (*p* < 0.001 for IGA, 0.003 for open comedone, 0.004 for closed comedone, *p* < 0.001 for all other lesion counts). The group using azelaic acid was different from the other groups in terms of all variables (IGA values and all lesion counts were lower in this group). In pairwise comparisons, there was a difference between the groups using erythromycin and clindamycin in terms of the count of pustules, inflammatory lesions, and total lesions, as before treatment (these variables were more in the group using clindamycin, *p* = 0.001, 0.023 and 0.041, respectively). Details are given in Table 1.

**FIGURE 1:**
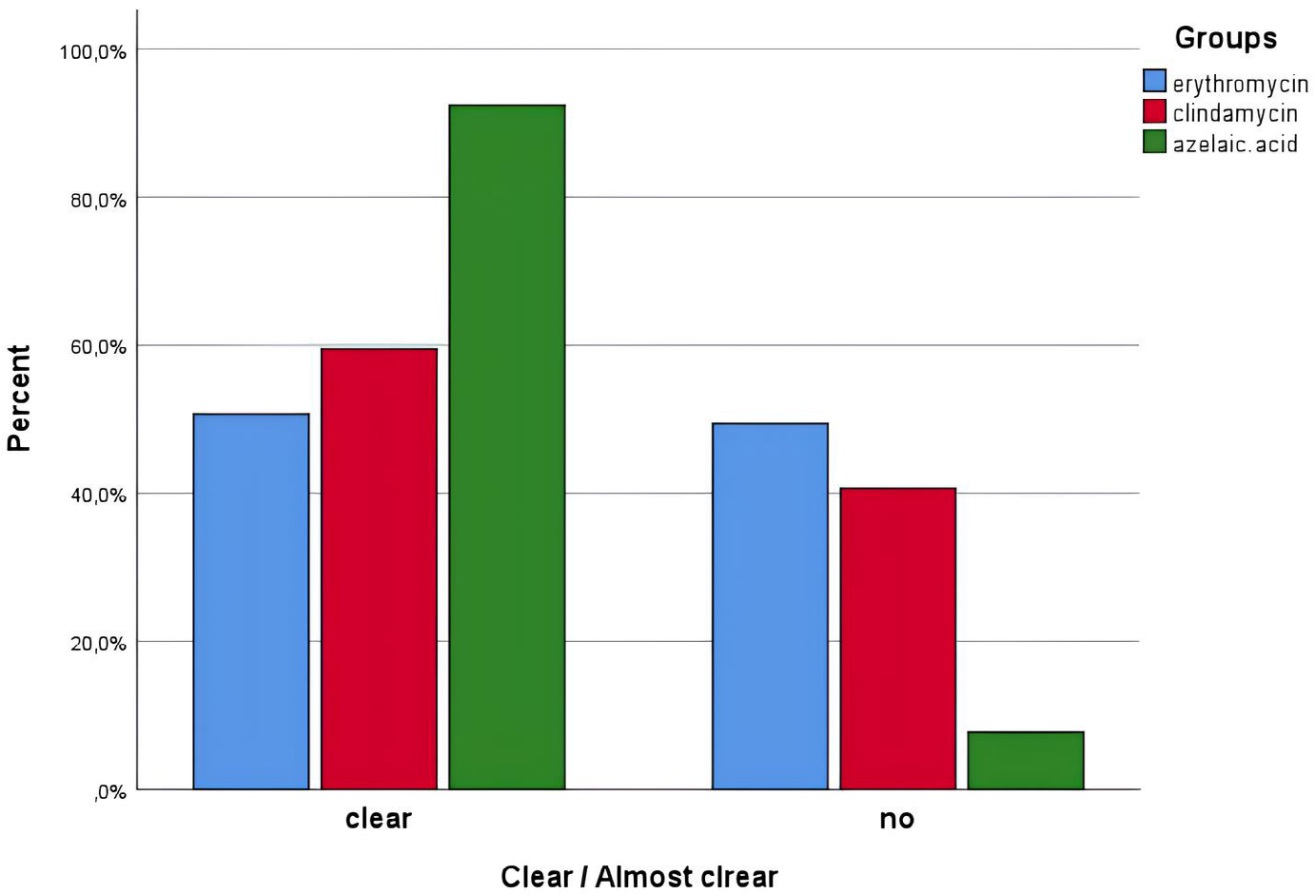
Percentages of recovered (IGA ≤ 1) cases.

When the participants were divided into two according to their IGA values at the end of the treatment: those with 0 and 1 as “clear/almost clear,” and the others as “others,” a statistically significant difference was found between the groups in this regard (clearance rates in the group using azelaic acid were much higher than in the other groups, *p* = 0.001). When the groups using erythromycin and clindamycin were compared among themselves in this respect, no significant difference was found (*p* = 0.255). Details are given in Figure 1.

**FIGURE 2:**
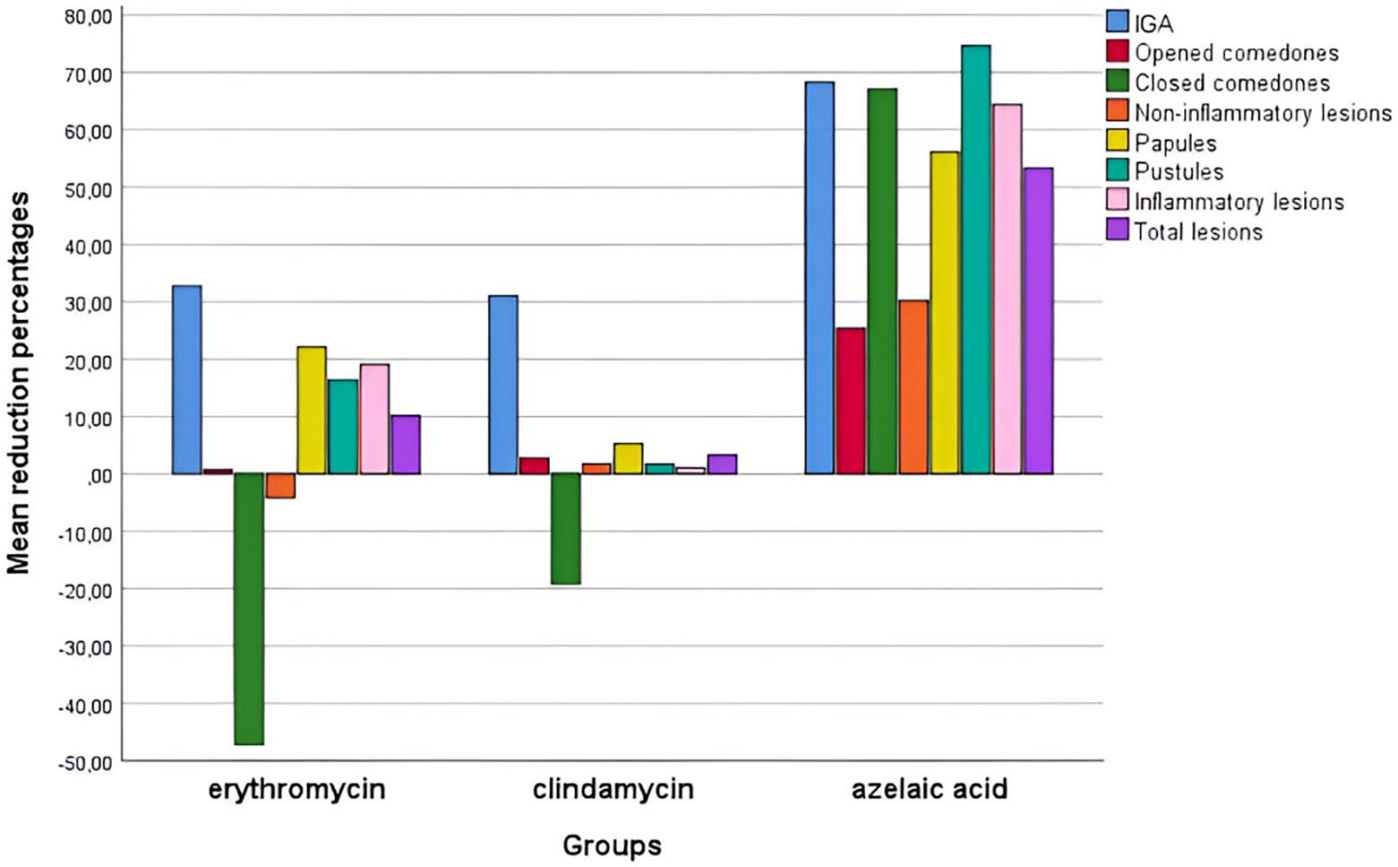
Improvement percentages by groups.

Since there were differences in some lesion numbers between the groups before treatment, the percentage changes in IGA values and lesion numbers were calculated in order to evaluate the changes after treatment more meaningfully. Before this, while the improvement percentages were investigated, values with a zero count were investigated both before and after the treatment, and it was seen that this situation was only in closed comedones. When calculating values for closed comedones, patients with zero values both before and after treatment were excluded from the calculation because the number of lesions in these patients neither increased nor decreased. In this case, the recovery percentage cannot be given a plus or minus number, and we thought that it would not be right to call this recovery percentage zero because it is not meaningful to say that a patient who did not have a closed comedone before the treatment and did not show an increase after the treatment did not improve or worsen. When the three groups were compared in terms of percentage changes in IGA and lesion counts, a statistically significant difference was found for all variables (*p* < 0.001 for all IGA and lesion counts). The group using azelaic acid was by far better in this regard, and the improvement rates were significantly higher in this group compared to the other two groups. At the same time, the two groups using erythromycin or clindamycin were compared among themselves in terms of these variables, and while there was no difference between these groups in terms of improvement rates in IGA levels, open and closed comedones, papules, and total lesion numbers (*p* = 0.401, 0.052, 0.291, 0.518 and 0.527, respectively), there was a difference in the improvement rates of non‐inflammatory lesions, pustules, and inflammatory lesions (*p* = 0.021, < 0.001 and 0.036, respectively). When we look at the data in Table 1, we see that closed comedones increased in both groups, without statistical difference, more in the group using erythromycin. When the percentages of improvement in non‐inflammatory lesions are examined, it is seen that there is a minimal improvement in those using clindamycin, whereas on the contrary, the lesions increase in those using erythromycin. Compared to those using erythromycin, there was minimal improvement in the number of pustules and inflammatory lesions in those using clindamycin. Details are given in Table 1 and Figure 2.

When the groups were compared in terms of whether side effects were observed or not, no statistically significant difference was found (*p* = 0.093). In patients with only side effects, it was also examined whether there was a difference in the types of side effects seen between the three groups (erythema, itching and flaking/desquamation side effects were mentioned in the patient files in the study) and a difference was found (*p* = 0.003); this difference was due to the clindamycin group (*p* = 0.013 between erythromycin and clindamycin, 0.002 between clindamycin and azelaic acid, 0.236 between erythromycin and azelaic acid). The itching side effect was only seen in patients using clindamycin. Details are given in Table 2.

**TABLE 2:** Side effects seen in patients.

*n*/%	Erythromycin (*n* = 75)	Clindamycin (*n* = 96)	Azelaic acid (*n* = 26)	*p*
No	71/94.7%	88/91.7%	21/80.8%	0.093
Yes	4/5.3%	8/8.3%	5/19.2%	
*Erythema*	*3/75%*	*1/12.5%*	*5/100%*	***0.003***
*Itching*	*0/0%*	*7/87.5%*	*0/0%*	
*Flaking/desquamation*	*1/25%*	*0/0%*	*0/0%*	

*Note:* Bold letters in the table indicate significant results.

When the groups were compared in terms of patient satisfaction, a significant difference was found (*p* < 0.001). While the percentage of those who were very satisfied was very low in the group using clindamycin and much higher in the group using azelaic acid, the number of those who were dissatisfied was lower in the patients using azelaic acid. Additionally, when compared in pairs, a difference was found between all groups in terms of satisfaction levels (*p* = 0.03 for groups using erythromycin and clindamycin, < 0.001 for clindamycin and azelaic acid, 0.003 for erythromycin and azelaic acid). When only dissatisfied patients were evaluated, it was also examined whether there was a difference between the groups in terms of the reasons for dissatisfaction (side effects or insufficient effects were mentioned in the patient files included in the study) and a statistically significant difference was found (*p* = 0.045). The difference was due to the group using azelaic acid, in which all those who were dissatisfied stated that they were dissatisfied due to the side effect (all were erythema, Table 2). There was no difference in this respect in the pairwise comparison between the groups using erythromycin and clindamycin (*p* = 1). Details are given in Table 3.

**TABLE 3:** Satisfaction status of patients.

*n*/%	Erythromycin (*n* = 75)	Clindamycin (*n* = 96)	Azelaic acid (*n* = 26)	*p*
Very satisfied	8/10.7%	1/1%	10/38.5%	**< 0.001**
Satisfied	49/65.3%	59/61.5%	14/53.8%	
Dissatisfied	18/24%	36/37.5%	2/7.7%	
*Side effects*	*4/22.2%*	*8/22.2%*	*2/100%*	***0.045***
*Insufficient effects*	*14/77.8%*	*28/77.8%*	*0/0%*	

*Note:* Bold letters in the table indicate significant results.

## 4 Discussion

The use of topical antibiotics alone is not recommended by the authors based on the issue of resistance; combination treatments with retinoids or benzoyl peroxide are generally emphasized [5]. However, due to the lack of topical acne options that can be used during pregnancy, topical antibiotics can also be used.

The Food and Drug Administration (FDA) divides drugs into A, B, C, D, and X in terms of their usability and potential risks during pregnancy (Table 4). Systemic absorption of topical clindamycin is very low and is considered safe in all trimesters of pregnancy [6, 7, 8, 9]. Due to the possibility of pseudomembranous enterocolitis, a history of gastrointestinal disease should be questioned and used with caution. Topical erythromycin is also safe in all trimesters [6, 7, 8, 9], but the resistance rate was found to be high [10]. In studies conducted on azelaic acid, which, in addition to its anti‐microbial effect (it has a bacteriostatic effect on both aerobic and anaerobic bacteria), also has anti‐inflammatory and comedolytic effects [11], and is also known to block 5 alpha reductase, no fetal side effects were observed after use during pregnancy [6, 7, 12] and its use twice a day has been found safe [6, 13].

**TABLE 4:** Drugs commonly used in acne treatment and their FDA categories.

	A	B	C	D	X
Definition	There are sufficient studies and no risk has been demonstrated	Animal studies have not shown a risk, but there are not enough human studies	Side effects have been shown in animal studies, but there are not enough human studies (use may be considered if there is significant benefit)	Fetal risks have been demonstrated in humans	Animal and human studies have detected fetal anomalies (use should not be considered for any benefit)
Medicines					
*Topicals*					
Clindamycin		✓			
Erythromycin		✓			
Azelaic acid		✓			
Benzoyl peroxide			✓		
Sodium sulfocetamine			✓		
Retinoids			✓ (tretinoin, adapalene)		✓ (tazarotene, contraindicated)
*Oral medications*					
Tetracycline				✓(Contraindicated in the 2nd and 3rd trimesters)	
Azithromycin		✓			
Clindamycin		✓			
Isotretinoin					✓ (contraindica‐ted)

Pazoki et al. investigated the efficacy, patient satisfaction rates, and side effect profiles of the combination of 5% AA +2% erythromycin and 20% AA and 2% erythromycin in acne vulgaris in a 12‐week, multicenter, randomized, double‐blind study of 147 patients with moderate‐to‐severe acne [14]. The female patients in the study were not pregnant. The authors found that combination therapy was more effective on the number of lesions (*p* < 0.05); side effects were less common in this group (27% vs. 45%–54%); and patient satisfaction was higher (80% satisfied/very satisfied).

Pazoki‐Toroudi et al. conducted a multicenter, randomized, and double‐blind study in a total of 88 male and 62 non‐pregnant female patients, and compared topical 2% clindamycin (32 males and 18 females), 5% azelaic acid (27 males and 23 females) and the combination of these two drugs (29 men and 21 women) in terms of effectiveness and side effects during 12 weeks [15]. As a result of the study, a significant decrease was observed in the number of total lesions between before and after treatment in both groups using clindamycin and azelaic acid (percentage reduction 46.89 ± 3.62 and 34.94 ± 2.67, *p* < 0.05 and < 0.01, respectively). In our study, while a slight improvement was observed in the group using clindamycin, a greater decrease in the number of lesions was observed in the group using azelaic acid. In this study, improvement percentages were not compared between the groups using azelaic acid and clindamycin. At the end of the study, they found the percentage improvement in the acne severity index to be 47.73% ± 6.62% in the clindamycin group and 32.46% ± 5.27% in the azelaic acid group, but the score improvement percentages for these two groups were not compared. In our study, we found a decrease in IGA levels, as in this study, but in our study, the decrease was less in the clindamycin group and more in the azelaic acid group. The percentage decreases in the number of papules, pustules, and comedones in the clindamycin and azelaic acid groups were 53.03 ± 3.26 versus 28.00 ± 3.21, 42.10 ± 4.41 versus 32.39 ± 3.22, and 45.54 ± 4.29 versus 44.43 ± 4.34, respectively. In our study, unlike this study, we observed a lower improvement in the number of papules, pustules, and comedones in the clindamycin group. In our study, while the improvement in comedones was lower in the azelaic acid group compared to this study, the percentage of improvement in the number of papules and pustules was higher. As can be seen, the effects of clindamycin and azelaic acid on acne treatment are contrasting between this study and ours. This may be due to several factors:

1. All participants in our study were pregnant women; however, this study included both male and female participants, and the women were not pregnant.
2. In our study, clindamycin was 1% and azelaic acid was 20%; while in this study, clindamycin was 2% and azelaic acid was 5%.

In the study, patient satisfaction levels were divided into five levels: 0—very dissatisfied, 1—dissatisfied, 2—moderately satisfied, 3—satisfied, and 4—very satisfied. In the group using clindamycin, the number of those who were dissatisfied and very dissatisfied was 6 (12%), the number of those who were moderately satisfied and satisfied was 35 (70%), and the number of those who were very satisfied was 9 (18%). In the group using azelaic acid, the number of those who were dissatisfied and very dissatisfied was 8 (16%), the number of those who were moderately satisfied and satisfied was 37 (74%), and the number of those who were very satisfied was 5 (10%). In our study, unlike this study, the percentage of those who were dissatisfied was higher in the group using clindamycin, the percentage of those who were satisfied and very satisfied was lower, and in the group using azelaic acid, the percentage of those who were dissatisfied and satisfied was lower, and the percentage of those who were very satisfied was higher. While in the group using clindamycin, 6 (12%) people had flaking, 3 (6%) had dry skin, 4 (8%) had erythema, and 3 (6%) had itching, these numbers were 4 (8%), 5 (10%), 3 (6%), and 4 (8%), respectively, in the group using azelaic acid. In our study, we found erythema and scaling to be lower and itching to be more in the clindamycin group, and we found itching and scaling to be lower and erythema to be more in the azelaic acid group. In this study, no comparison was made between the clindamycin and azelaic groups in terms of any of the side effect variables given above (comparisons were made pairwise between only clindamycin or only the azelaic acid group and the combination group). In this study, the authors evaluated the efficacy and side effect profiles of azelaic acid and clindamycin, as well as the efficacy, satisfaction, and side effect profile of the combination of these two drugs. In the study, lesion reduction in the group using combination was higher than in the groups using AA and clindamycin (63% vs. 35%–47%; *p* < 0.01). At the same time, the satisfaction rate was found to be higher in the group using the combination (75%–86% satisfied/very satisfied patients). The authors found no significant difference in side effects between the combination group and the other groups using single drugs.

Gollnick et al. evaluated the results of two previous double‐blind studies comparing 15% azelaic acid versus 5% benzoyl peroxide in 351 mild‐to‐moderate acne patients and 15% azelaic acid versus 1% clindamycin in 229 mild‐to‐moderate acne patients [16]. While in the first study, a 70% improvement in the number of papules and pustules and a 60% improvement in the number of comedones was observed in the group using azelaic acid, in the second study, these rates were 71% and 57%, respectively. While we found similar improvement rates in the number of pustules in the group using azelaic acid in our study, the improvement rates we found in papules and comedones were lower than in this study. While in the first study, 6.3% skin dryness, 8.6% irritation symptoms, 2.3% flaking, 11.9% burning sensation, and 6.3% itching were observed in the group using azelaic acid, in the other study, these numbers were 8.8% (skin dryness and flaking together), 10.5%, 11.4%, and 8.8%, respectively. In our study, we observed less flaking/desquamation and itching in the group using azelaic acid.

Our results support more extensive evidence for the usefulness of azelaic acid in acne therapy. More recent studies demonstrate enhanced efficacy and resistance suppression by combining lower‐strength AA (5%) with antibiotics (e.g., erythromycin or clindamycin) compared with monotherapy [14, 15]. Even though our trial examined AA monotherapy in pregnancy, these findings anticipate future research into AA‐containing combinations will optimize results while minimizing resistance problems.

## 5 Conclusion

Considering the results of our study, we see that azelaic acid is more successful in the treatment of mild to moderate acne vulgaris during pregnancy, even though all three topical drugs are in pregnancy category B, when evaluated in terms of both effectiveness (decrease in IGA scores and lesion numbers) and patient satisfaction. At the same time, it is noteworthy that there were no side effects that required discontinuation of the drug or treatment. In addition, since the use of topical antibiotics alone highlights the issue of resistance, it is not supported by the authorities, and it is recommended to use them in combination with either retinoids, benzoyl peroxide, or azelaic acid. The studies mentioned in the discussion show that the combination of azelaic acid and antibiotics is more effective than azelaic acid or antibiotic treatment alone. AA‐antibiotic combinations must be investigated in pregnant populations in the future, as non‐pregnant evidence illustrates such combinations can exhibit greater efficacy at reducing resistance.

Therefore, azelaic acid has a dual action (anti‐inflammatory and antimicrobial); it is a good option in the treatment of mild to moderate acne seen during pregnancy.

### 5.1 Limitations

Since before and after photos were not available in the patient medical records, satisfaction was evaluated only on a patient basis; it was not possible for impartial dermatologists to evaluate the photos. In the study, patient records from a single center were investigated, so the results cannot be generalized to the society or other countries (larger patient numbers need to be reached and multicenter studies need to be conducted). The small number of patients in the azelaic acid group negatively affects statistical power; this risks Type II error (failure to detect true effects) and reduces generalizability.

## Ethics Statement

The study was approved by the clinical trials ethics committee of Hitit University (approve date 11.02.2025, approval number 2025‐21).

## Conflicts of Interest

The author declares no conflicts of interest.

## Supporting information


**Table a.** Cross‐Study Efficacy Comparison.
**Table b**. Patient Satisfaction.

## Data Availability

The data that support the findings of this study are available from the corresponding author upon reasonable request.
