# Supplementary material for: Retrospective Analysis of Efficacy and Side Effects of Topical 4% Erythromycin Versus 1% Clindamycin Versus 20% Azelaic Acid During Pregnancy
Source: J Cosmet Dermatol. 2025 Aug 28;24(9):e70410. doi: 10.1111/jocd.70410 (PMC12391849; doi:10.1111/jocd.70410)
Supplement: Supplementary file 1 — Table a. Cross‐Study Efficacy Comparison. Table b. Patient Satisfaction. [file JOCD-24-e70410-s001.docx]

**Table a: Cross-Study Efficacy Comparison**

| **Treatment** | **Our Study** (IGA Improvement) | **Pazoki et al. (15)** (Lesion Reduction) |
| --- | --- | --- |
| Azelaic Acid (AA) | 68.3% | 32–35% (monotherapy) |
| AA + Antibiotic | N/A | 63–65% (combo) |

**Table b: Patient Satisfaction**

| **Group** | **Our Study** (% Very Satisfied) | **Pazoki et al. (15)** (% Satisfied/Very Satisfied) |
| --- | --- | --- |
| Azelaic Acid (AA) | 38.5% | 30–50% (monotherapy) |
| AA + Antibiotic | N/A | 75–86% (combo) |
